# Structure–function models for estimating retinal ganglion cell count using steady-state pattern electroretinography and optical coherence tomography in glaucoma suspects and preperimetric glaucoma: an electrophysiological pilot study

**DOI:** 10.1007/s10633-022-09900-z

**Published:** 2022-09-26

**Authors:** Derek Orshan, Andrew Tirsi, Hosam Sheha, Vasiliki Gliagias, Joby Tsai, Sung Chul Park, Stephen A. Obstbaum, Celso Tello

**Affiliations:** 1 Department of Ophthalmology, Larkin Community Hospital, Miami, FL USA; 2 Department of Ophthalmology, Manhattan Eye, Ear, and Throat Hospital, Northwell Health, 210 E 64th St, 8th Floor,, New York, NY 10065 USA; 3 Donald and Barbara Zucker School of Medicine at Hofstra/Northwell, Hempstead, NY USA; 4 Diopsys Inc, Pine Brook, NJ USA

**Keywords:** Glaucoma, PERG, Retinal ganglion cell, OCT, Generalized linear mixed model

## Abstract

**Purpose:**

To derive and validate structure–function models for estimating retinal ganglion cell (RGC) count using optical coherence tomography (OCT) and steady-state pattern electroretinography (ssPERG) parameters in glaucoma suspects (GS) and preperimetric glaucoma (PPG).

**Methods:**

In this prospective cross-sectional study, 25 subjects (50 eyes) were recruited at the Manhattan Eye, Ear, and Throat Hospital. Subjects underwent comprehensive eye examinations, OCT, standard automated perimetry (SAP), and ssPERG testing. Eyes were divided into three groups based on the Global Glaucoma Staging System: healthy (*N* = 30), GS (*N* = 10), and PPG (*N* = 10) eyes. The combined structure–function index (CSFI), which estimates retinal ganglion cell count (eRGC_CSFI_) from SAP and OCT parameters, was calculated in each study subject. Two prediction formulas were derived using a generalized linear mixed model (GLMM) to predict eRGC_CSFI_ from ssPERG parameters, age, and average retinal nerve fiber layer thickness (ARNFLT) in 30 eyes selected at random (training group). GLMM predicted values were cross-validated with the remaining 20 eyes (validation group).

**Results:**

The ARNFLT, ssPERG parameters magnitude (Mag) and magnitudeD (MagD), and eRGC_CSFI_ were significantly different among study groups (ANOVA *p* ≤ 0.001). Pearson correlations demonstrated significant associations among ARNFLT, ssPERG parameters, and eRGC_CSFI_ (*r*^2^ ≥ 0.31, *p* < 0.001). Two GLMMs predicted eRGC_CSFI_ from Mag (eRGC_Mag_) and MagD (eRGC_MagD_), respectively, with significant equations (*F*(3,18), *F*(3,19) ≥  58.37,* R*^2^ = 0.90, *p* < 0.001). eRGC_Mag_ and eRGC_MagD_ in the validation group (*R*^2^ = 0.89) correlated with eRGC_CSFI_ similarly to the training group. Multivariate pairwise comparisons revealed that eRGC_Mag_ and eRGC_MagD_ distinguished between healthy, GS, and PPG eyes (*p* ≤ 0.035), whereas independent Mag, MagD, and ARNFLT measures did not distinguish between GS and PPG eyes.

**Conclusion:**

This pilot study offers the first combined structure–function models for estimating RGC count using ssPERG parameters. RGC counts estimated with these models were generalizable, strongly associated with CSFI estimates, and performed better than individual ssPERG and OCT measures in distinguishing healthy, GS, and PPG eyes.

## Introduction

Glaucoma is a group of optic neuropathies characterized by optic nerve head and retinal nerve fiber layer (RNFL) thinning due to progressive retinal ganglion cell (RGC) dysfunction and death [1]. Most subtypes of glaucoma are painless and progress slowly, with RGC losses occurring before visual symptoms manifest [2]. Early identification of individuals at high risk of developing glaucoma therefore plays an important role in initiating medical intervention and improving quality of life [3].

The pattern electroretinogram (PERG), an electrophysiological test that measures RGC function [4, 5], was shown capable of detecting RGC dysfunction in glaucoma suspects (GS) and preperimetric glaucoma (PPG) [6–8]. Animal studies directly linked PERG losses to optic nerve head damage [9], and in humans, PERG losses preceded RNFL thinning [10], vision loss [11], and correlated with age-related RGC losses [12]. Recently, a method of PERG with excellent reproducibility and repeatability [13, 14], steady-state PERG (ssPERG), was developed, allowing for widespread utilization in practice [15–18]. Recent ssPERG studies investigated RGC dysfunction in PPG and found that ssPERG parameters correlated with glaucomatous changes in optic nerve head morphology [17].

The combined structure–function index (CSFI) is a mathematical algorithm that estimates RGC count (eRGC_CSFI_) from SAP and OCT parameters [19], and studies that utilized eRGC_CSFI_ found a strong correlation with histologically verified RGC count in humans postmortem [20, 21]. Clinically, longitudinal studies showed that eRGC_CSFI_ performed better than independent OCT and SAP parameters in distinguishing healthy from glaucomatous eyes [19, 20] and predicting glaucoma progression in GS [22]. Importantly, our previous investigation correlated ssPERG parameters to estimated RGC counts derived from the CSFI (eRGC_CSFI_) and demonstrated that eRGC_CSFI_ directly mediated the relationship between ssPERG parameters and average retinal nerve fiber layer thickness (ARNFLT). In other words, this study provided evidence that previously described correlations between ssPERG parameters and ARNFLT [18, 23] were likely attributable to their independent associations with RGC count [18].

This pilot study sought to determine if RGC count could be estimated through structure–function models that utilize OCT and ssPERG parameters. Such models could provide a new approach to estimating RGC count in GS and PPG.

## Methods

In this prospective cross-sectional study, a total of 25 consecutive subjects (50 eyes) were recruited from the Manhattan Eye Ear Throat Hospital ophthalmology practice and divided into three groups: healthy subjects, GS, and PPG, based on the Global Glaucoma Staging System (GGSS), which integrates the OCT Glaucoma Staging System [24] and Glaucoma Staging System 2 (GSS) [25] into a unified structure–function classification system [26]. Eyes that fell within the borderline range of the GGSS were categorized as GS eyes, whereas eyes within Stage 1 with predominant structural damage were classified as PPG eyes [25]. A total of five study participants had eyes that were classified into two different groups, and the remainder of study participants had both eyes within the same group. One subject had one eye as healthy and the other as GS, while the other four subjects had one eye in the GS group and the other eye in the PPG group. Participants underwent a complete ophthalmologic examination, including slit lamp biomicroscopy, Goldmann tonometry, standard automated perimetry (Humphrey Field Analyzer II, 24-2 SITA-Standard strategy), OCT (Carl Zeiss Meditec Inc., Dublin, CA, USA) and ssPERG (Diopsys Inc., Pine Brook, NJ, USA).

Participants were 20–80 years old and had a best-corrected visual acuity (BCVA) better or equal to 20/40 as measured by Snellen visual acuity testing at the time of enrollment. All participants had a documented and repeatable normal HFA 24-2 at the baseline visit. No participants were on intraocular pressure (IOP) lowering treatment at the time of enrollment. All participants with prior intraocular or posterior segment intraocular surgery, ocular trauma, or ocular or systemic conditions that may affect optic nerve head structure and/or function, except for uncomplicated cataract extraction with posterior chamber intraocular lens implant performed less than a year before enrollment, were excluded from this study. Two GS subjects and two age-matched healthy controls under the age of 40 were enrolled in this study. GS subjects had a strong family history of open-angle glaucoma and suspicious optic nerve head findings [18].

### Spectral-domain optical coherence tomography

Average retinal nerve fiber layer thicknesses (ARNFLT) were measured using the Optic Disk Cube protocol of a Cirrus spectral-domain optical coherence tomography (SD-OCT) version 6.0. The protocol scans a 6 × 6-mm area centered around the optic nerve head, collecting 200 × 200 axial scans containing 40,000 points. ARNFLT is measured segmentally in quadrants and clock hour sectors within a 3.46 mm region of interest [18, 27].

### Visual field testing

All patients underwent SAP testing using the HFA 24-2 protocol. Visual fields with more than 20% fixation losses, 24-2 mean deviation (MD) < −2 dB and glaucoma hemifield test (GHT) outside normal limits, false-negative errors, and false-positive errors were excluded. Using HFA SITA 24-2 results, only participants with visual fields corresponding to stage 0 (no visual field losses) following the GSS were considered [18, 25].

### ssPERG testing

The ssPERG in this study follows the PERGLA protocol established by Porciatti et al. [15], which was developed to simplify PERG-assisted glaucoma screening. The PERGLA protocol adds filters and amplifiers to ssPERG recordings to achieve an amplitude and signal-to-noise ratio adherent to the International Society for Clinical Electrophysiology of Vision (ISCEV) standards [14, 18, 28–30].

The ssPERG was recorded using Diopsys® NOVA-PERG (Diopsys, Inc. Pine Brook, New Jersey, USA), and was described previously [17, 18]. Tests were performed in a dark room to standardized environment luminance, free of visual, and audible distractions. Subject’s forehead skin was cleaned using NuPerp® Skin Prep Gel (Weaver and Company, CO, USA) and the lower eyelids using OCuSOFT ® Lid Scrub Original (OCuSOFT® Inc., Rosenberg, TX, USA) to ensure good and stable electrical activity. Disposable hypoallergenic skin sensors silver/silver chloride ink (Diopsys® proprietary Skin Sensor) were applied on the lower lid of both eyes, at the lid margin and avoiding eyelashes. One ground sensor (Diopsys® EEG electrode) was applied in the central forehead area with a small amount of conductive paste (Ten20®, Weaver and Company), and cables from the Diopsys NOVA device were connected to the electrodes. A total of 3 electrodes were used per test per patient. Subjects were fitted with the appropriate correction for a viewing distance of 24 inches and were instructed to fixate on a target at the center of the monitor in front of them [17, 18].

The stimulus was presented on a gamma-corrected Acer 192 V176BM 17-inch monitor, having a refresh rate of 75 frames/s. Luminance output over time was verified using a luminance meter MavoSpot 2 USB (Gossen, GmbH, Nuremberg; Germany). The pattern stimulus consisted of black/white alternating square bars, reversing at 15 reversals/s (rps) with a duration of 25 s for high contrast [HC 85%] and 25 s for low contrast [LC 75%] for a total of 50 s per eye. The stimulus field subtends a visual angle of 1439.90 arc min. Each bar will subtend 22.49 arc min, for a total of 64 bars. A red target subtending 50.79 arc min was used as a fixation target and was centered on the stimulus field [18]. The luminance of the white bars for 85 and 75% contrast was 204 cd/m2 and the luminance for black was 20.5 and 52.5 cd/m2 yielding a mean luminance of 112.3 and 128.2 cd/m2, respectively. All recorded signals underwent band filtration (0.5–100 Hz), amplification (gain = 20,000), and averaging at least 150 frames. The signal was sampled at 1920 samples per second by an analog to digital (A/D) converter. The voltage range of the (A/D) converter was programmed between −5 V and + 5 V. Sweeps contaminated by eye blinks or gross eye saccades were automatically rejected if they exceeded a threshold voltage of 50 μV, and these sections were identified as artifacts in the report. Synchronized single-channel electroretinography was recorded, generating a time series of 384 data points per analysis frame (200 ms) [18]. An automatic fast Fourier transformation was applied to the PERG waveforms to isolate the desired component at 15 rps. Other frequencies, such as those originating from eye muscles, were rejected. The PERG test results were saved in an SQL database and presented in a report form to be used for analysis. For every subject, four pre-programmed full "contrast sensitivity 214 protocols" were performed sequentially, which consisted of two 25-s recordings for each eye: first with high-contrast (85%) diffuse retinal stimulation, then with low-contrast (75%) pattern stimulation. The device collected 5 frames of data per second, totaling 125 frames of data, and the first 10 frames (2 s) of data were discarded [17, 18].

For each eye, three PERG measurements (Magnitude [Mag], MagnitudeD [MagD], and MagD/Mag ratio) were calculated. Mag (µV) represents the amplitude of the signal strength at the specific reversal rate of 15 Hz in the frequency domain, while MagD (µV) represents an adjusted amplitude of the PERG signal impacted by phase variability throughout the waveform recording. MagD is considered to equal the Mag, which was altered by phase change, and therefore it is also considered to reflect phase consistency [17, 18]. MagD/Mag ratio data were excluded due to a lack of impact on the findings in this study.

A recording where the phase of the response is consistent will produce a MagD value close to that of the Mag, whereas a recording where the phase of the response varies will produce a MagD value lower than that of Mag. This is because averaging responses that are out-of-phase with each other will cause some degree of cancellation [18]. Please see reference 18 for a detailed explanation of the relationship between Mag and MagD.

These parameters are repeatable, reproducible, and sufficiently reliable in clinical practice [14, 18]. Results were also presented in a color-coded system, like “traffic light system,” with green color—showing the results being within reference range, yellow—represented values within borderline reference range, and red color-represented results outside reference range [18].

### Estimating RGC counts with the combined structure–function index

Estimated RGC counts were calculated with the CSFI in accordance with formulas derived by Meideros et al. [19, 31]. The first step involves estimating RGC count using SAP sensitivity (*s*) values in dB at a given eccentricity (*ec*). The following formulas were used to determine SAP-derived RGC counts (SAPrgc):

$$m = \left[ {0.54\left( {ec*1.32} \right)} \right] + 0.9$$

$$b = \left[ { - 1.5 \left( {ec + 1.32} \right)} \right] {-} 14.8$$

$$gc = \frac{{\left( {s - 1} \right) - b}}{m} + 4.7$$

1 $${\text{SAPrgc}} = \sum 10^{{\left( {gc*0.1} \right)}}$$

In the above formulas, *m* and *b* represent the slope and intercept, respectively, of a linear function that relates ganglion cell counts (gc) to s at a given ec [19]. All RGC densities were uniform within each individual test location corresponding to 6 × 6 degrees of visual space.

SD-OCT-derived RGC counts (OCTrgc) were determined with the following formulas:

$$d = \left( { - 0.007 + {\text{age}}} \right) + 1.4$$

$$c = \left( { - 0.26*{\text{MD}}} \right) + 0.12$$

$$a = {\text{average RNFLT*}}10870*d$$

2 $${\text{OCTrgc}} = 10^{{\left\{ {\left[ {\log \left( a \right)*10 - c} \right]*0.1} \right\}}}$$

In the above formulas, d corresponds to axonal density (axons/um^2^) and c is a correction factor that considers the degree of functional visual impairment in order to account for retinal nerve fiber layer remodeling in advanced disease [19]. eRGC_CSFI_ was obtained using the following formula:

3 $${\text{eRGC}}_{{{\text{CSFI}}}} = \left( {1 + \frac{{{\text{MD}}}}{30}} \right)*{\text{OCTrgc}} + \left( { - \frac{{{\text{MD}}}}{30}} \right)*{\text{SAPrgc}}$$

Further rationale has been previously described in detail by Medeiros et al. [19].

### Estimating RGC count with generalized linear mixed models and cross-validation

Study participants were subdivided into two groups generated at random: a training group (*N* = 30, 60% of study participants) and a validation group (*N* = 20, 40% of study participants). Two generalized linear mixed models (GLMMs) were applied to the training sample to predict eRGC_CSFI_. eRGC_Mag_ and eRGC_MagD_ were then calculated for each study subject by applying the GLMM formula to all eyes within our cohort. The GLMM used to generate eRGC_Mag_ included Mag, ARNFLT, and age as prediction (independent) variables, whereas the eRGC_MagD_ model included MagD, ARNFLT, and age. Within-subject inter-eye correlations were accounted for by assigning both eyes of each study subject as repeated measures to allow for separate units of analysis, and by applying an unstructured covariance structure (with random intercept) to both GLMMs [32, 33]. eRGC_Mag_ and eRGC_MagD_ were cross-validated with validation group estimates. Model fitness was determined using *R*^2^, standard error (SE), and *F*-values.

### Statistical analysis

Mean and standard deviation values were determined for Mag and MagD, HFA SITA standard (24-2) tests, and ARNFLT. Descriptive statistics among healthy, borderline, and early glaucoma eyes were conducted using ANOVA. Differences between groups were analyzed using Games-Howell post hoc multivariate pairwise comparisons to account for within-subject inter-eye correlations [32, 34] and an uneven distribution of eyes among groups [35]. Associations among dependent and independent variables were analyzed using Pearson correlations.

## Results

In this study, ANOVA revealed that 24–2 MD, eRGC_CSFI_, and all variables used in our GLMMs (age, ARNFLT, Mag, and MagD) were significantly different among study groups (*p* < 0.001, Table 1). There was no significant difference in gender or IOP among study groups (Table 1). Pearson correlations revealed significant associations among eRGC_CSFI_ and age, ARNFLT, Mag, and MagD (*R*^2^ ≥ 0.31, *p* < 0.001) (Table 2).

**Table 1:** Summary of characteristics from an ANOVA among study participants within study groups (*N* = 50 eyes)

	Healthy eyes	GS eyes	PPG eyes	ANOVA *p*-value	Effect size
	Mean (± SD)	Mean (± SD)	Mean (± SD)		
*N* (eyes)	30	10	10	–	–
Age (years)*	49 (± 12)	50 (± 19)	73 (± 16)	< 0.001	0.365
Female (%)	33%	50%	30%	0.59	0.022
IOP (mmHg)	17.13 (± 3.85)	17.70 (± 4.14)	16.80 (± 3.12)	0.86	0.006
24-2 MD (dB)*	0.10 (± 1.06)	− 0.45 (± 0.94)	− 1.06 (± 1.72)	0.030	0.138
ARNFLT (μM)*	96.80 (± 7.95)	85.00 (± 5.75)	79.2 (± 5.35)	< 0.001	0.536
Mag (μV)*	1.95 (± 0.64)	1.35 (± 0.32)	1.25 (± 0.28)	< 0.001	0.355
MagD (μV)*	1.72 (± 1.06)	1.09 (± 0.34)	0.79 (± 0.67)	< 0.001	0.364
eRGC_CSFI_*	1,076,635 (± 127,284)	912,667 (± 127,284)	702,679 (± 103,847)	< 0.001	0.620

*SD* standard deviation*; IOP* intraocular pressure*; MD* mean deviation*; ARNFLT* average retinal nerve fiber layer thickness*; Mag* magnitude*; MagD* magnitudeD*; RGC*_*CSFI*_ combined structure–function index estimated retinal ganglion cell count

* ANOVA *p* < 0.001

**Table 2:** Pearson correlations among GLMM variables and eRGC_CSFI_

	*R*^2^	*p*-value
Age (Years)	0.57	< 0.001
ARNFLT	0.72	< 0.001
Mag	0.31	< 0.001
MagD	0.34	< 0.001

*GLMM,* generalized linear mixed model; *ARNFLT* average retinal nerve fiber layer thickness (μM); *Mag* magnitude (μV); *MagD* magnitudeD (μV)*; eRGC*_*CSFI*_ combined structure–function index estimated retinal ganglion cell count

Independent sample *t*-tests with variance not assumed were used to compare means in the randomly generated training (*N* = 30) and validation (*N* = 20) groups and revealed no significant difference in age, Mag, MagD, ARNFLT, or eRGC_CSFI_ between groups (Table 3). There were significantly less females in the validation group (23%) than in the training group (55%, *p* = 0.028), however, the training group was 55% female (Table 3).

**Table 3:** Summary of characteristics from an independent samples* t*-test with variance not assumed between training and validation groups (*N* = 50 eyes)

	Training group	Validation group	
	Mean (± SD)	Mean (± SD)	*p*-value
*N* (eyes)	30	20	–
Age (years)	52 (± 15)	56 (± 16)	0.39
Female (%)*	55%	23%	0.028
IOP (mmHg)	17.07 (± 4.20)	17.35 (± 2.92)	0.80
24–2 MD (dB)	− 0.19 (± 1.13)	− 0.32 (± 1.47)	0.73
ARNFLT (μM)	93.27 (± 10.69)	87.40 (± 8.66)	0.05
Mag (μV)	1.81 (± 0.68)	1.51 (± 0.48)	0.09
MagD (μV)	1.51 (± 0.71)	1.25 (± 0.58)	0.17
eRGC_CSFI_	1,009,793 (± 190,734)	907,936 (± 173,978)	0.06

*SD,* standard deviation; *IOP* intraocular pressure; *MD* mean deviation*; ARNFLT* average retinal nerve fiber layer thickness; *Mag* magnitude; *MagD,* magnitudeD*; RGC*_*CSFI*_ combined structure–function index estimated retinal ganglion cell count. * p < *0.05*

Both GLMMs used to generate eRGC_Mag_ and eRGC_MagD_ from training group eyes resulted in significant equations (*F*(3,18)*, F*(3,19) ≥  58.37*, R*^2^ = *0.90, p* < 0.001). eRGC_Mag_ and eRGC_MagD_ can be obtained through the following formulas (Table 4):

$${\text{eRGC}}_{{{\text{Mag}}}} = 401,342 - \left( {6268*{\text{Age}}} \right) + \left( {8899*{\text{ARNFL}}T} \right) + \left( {58,610*{\text{Mag}}} \right)$$

$${\text{eRGC}}_{{{\text{MagD}}}} { = }405,529 - \left( {6092*{\text{Age}}} \right) + \left( {9019*{\text{ARNFLT}}} \right) + \left( {53,493*{\text{MagD}}} \right)$$

**Table 4:** Results of two generalized linear mixed models (unstructured covariance) for predicting eRGC_CSFI_

Prediction variables	Coefficient	SE	*F*	*p-value*
Magnitude model (eRGC_Mag_)				
Constant	401,341.71	155,818.98	–	0.019
Age (Years)	− 6268.04	991.91	39.93	< 0.001
ARNFLT (μM)	8899.32	1615.10	30.36	< 0.001
Magnitude (μV)	58,610.30	24,744.92	5.61	0.027
MagnitudeD model (eRGC_MagD_)				
Constant	405,528.50	157,900.55	–	0.019
Age (Years)	− 6091.65	998.267	37.24	< 0.001
ARNFLT (μM)	9018.93	1631.04	30.58	< 0.001
MagnitudeD (μV)	53,492.96	24,090.70	4.93	0.027

Summary statistics	*R*^*2*^ (Training group)	*R*^*2*^ (Validation group)	*F* (Total)	*p-value*
eRGC_Mag_	0.90	0.89	58.861	< 0.001
eRGC_MagD_	0.90	0.89	58.371	< 0.001

*eRGC*_*CSFI*_*,* combined structure–function index estimated retinal ganglion cell count; *eRGC*_*Mag*_*,* magnitude model estimated retinal ganglion cell count; *eRGC*_*MagD*_ magnitudeD model estimated retinal ganglion cell count. *ARNFLT* average retinal nerve fiber layer thickness. Training group *N* = *30* eyes. Validation group *N* = *20* eyes

**Fig. 1:**
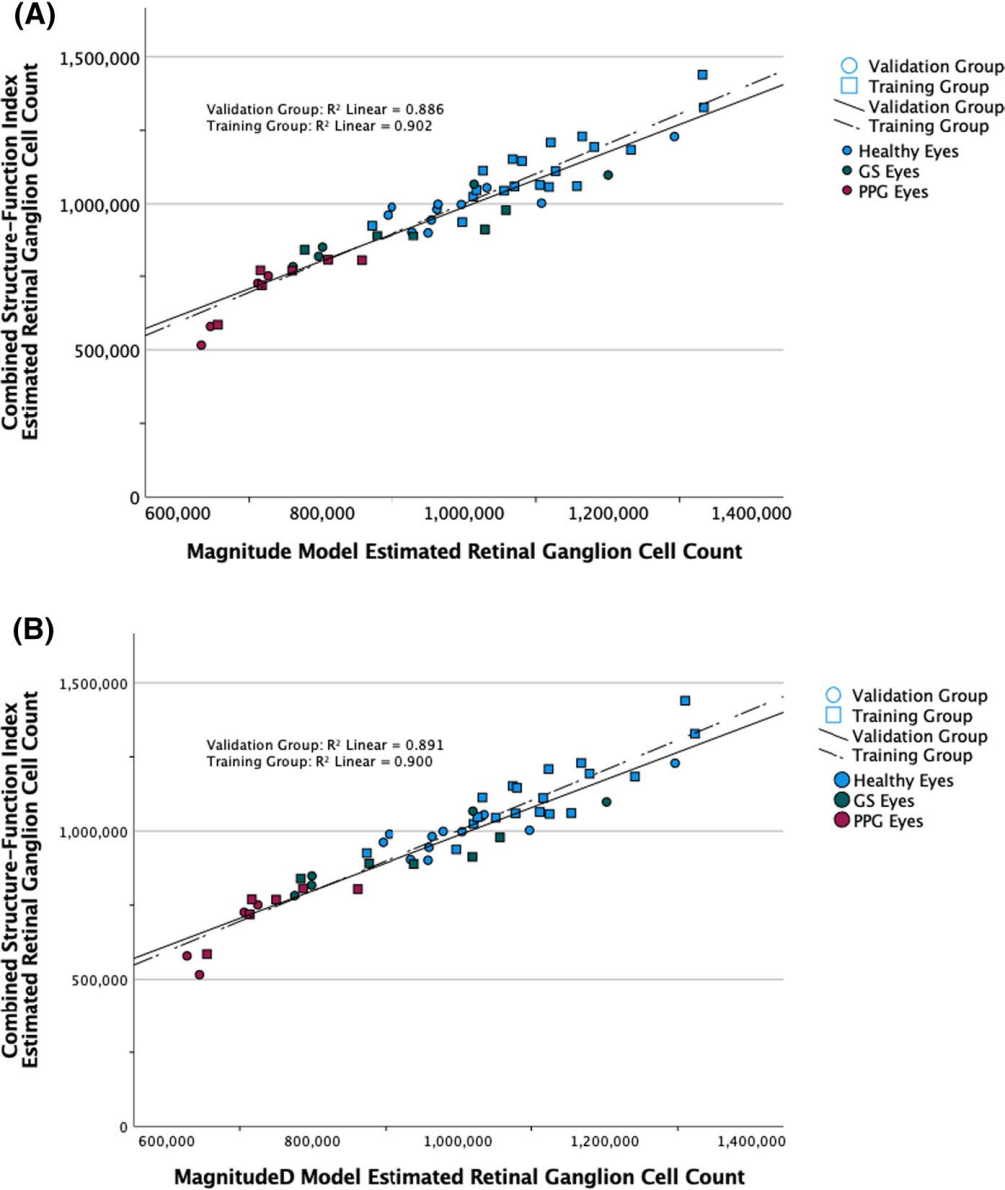
Scatterplot diagram of retinal ganglion cell counts estimated with the combined structure–function index (CSFI, *y*-axis) and a generalized linear mixed models (GLMM) of: **a** magnitude (Mag), average retinal nerve fiber layer thickness (ARNFLT), and age, and **b** magnitudeD (MagD), ARNFLT, and age (*X*-axis). Training group (squares, *N* = 30) data was used to train the GLMM. Validation group (circles, *N* = 20) data was used to cross-validate the GLMM. Blue: healthy eyes (*N* = 30); Green: borderline eyes (*N* = 14); Red: early glaucoma eyes (*N* = 6)

Training and validation group data demonstrated similar correlations among eRGC_CSFI_, eRGC_Mag_, and eRGC_MagD_ values (validation group *R*^2^ = 0.89 for both models) (Table 4). Scatterplot diagrams relating eRGC_CSFI_ to eRGC_Mag_ (Fig. 1a) and eRGC_MagD_ (Fig. 1b) are shown. Training group eyes are distinguished from validation group eyes by shape, and study groups are distinguished by color.

Games-Howell post hoc multivariate pairwise comparisons among study groups revealed that independent Mag, MagD, and ARNFLT measures distinguished healthy eyes from GS and PPG eyes (*p* ≤ 0.001) but did not distinguish GS from PPG eyes (*p* ≥ 0.08) (Table 5). 24–2 MD did not distinguish between any study groups. eRGC_Mag_, eRGC_MagD_, and eRGC_CSFI_ significantly distinguished between all three study groups (*p* ≤ 0.038). Mean differences in estimated RGC count were similar between GS and PPG eyes (≤ 209,988 RGCs) and between healthy and GS eyes (≤ 163,968 RGCs) (Table 5). All comparisons were similar between eRGC_Mag_ and eRGC_MagD_, suggesting that both ssPERG parameters can be incorporated into GLMMs without significantly affecting predictive values.

**Table 5:** Results from a Games-Howell post hoc multivariate pairwise comparison of study groups, accounting for inter-eye correlations

	Healthy eyes vs. GS eyes		Healthy eyes vs. PPG eyes		GS eyes vs. PPG eyes	
	Mean difference	*p*-value	Mean difference	*p*-value	Mean difference	*p*-value
Age^†‡^	0.60	1.00	23.60	< 0.001	23.00	0.011
IOP	0.57	0.92	0.33	0.96	0.90	0.85
24–2 MD	0.56	0.28	1.16	0.15	0.61	0.60
ARNFLT*^†^	11.80	< 0.001	17.60	< 0.001	5.80	0.08
Mag*^†^	0.60	0.001	0.69	< 0.001	0.10	0.76
MagD*^†^	0.63	0.001	0.93	< 0.001	0.30	0.14
eRGC_CSFI_ *^†‡^	163,968	0.002	373,956	< 0.001	209,988	< 0.001
eRGC_Mag_*^†‡^	143,880	0.038	345,229	< 0.001	201,349	0.005
eRGC_MagD_*^†‡^	143,690	0.035	352,102	< 0.001	208,412	0.003

*IOP,* intraocular pressure (mmHg); *MD* mean deviation (dB); *ARNFLT* average retinal nerve fiber layer thickness (μM); *Mag* transformed magnitude (μV); *MagD* transformed magnitudeD (μV); *RGC*_*CSFI*_ retinal ganglion cell count estimated with the combined structure–function index*; eRGC*_*Mag*_ retinal ganglion cell count estimated with Mag and ARNFLT; *eRGC*_*MagD*_ retinal ganglion cell count estimated with MagD and ARNFLT

* *p* < *0.05* for control eyes vs. glaucoma suspect (GS) eyes.^†^*p* < *0.05* for control eyes vs. preperimetric glaucoma (PPG) eyes. ^‡†^* p* < *0.05* for GS eyes vs. PPG eyes. Healthy eyes *(N* = *30),* glaucoma suspects *(N* = *10),* preperimetric glaucoma *(N* = *10)*

Receiver operating characteristics (ROC) curve analysis revealed that ARNFLT performed best among study variables (AUC = 0.89, *p* < 0.001) in distinguishing healthy eyes from GS eyes, whereas eRGC_Mag_ and eRGC_MagD_ performed the worst (AUC ≤ 0.78, *p* = 0.004). However, eRGC_CSFI_, eRGC_CSFI_, eRGC_CSFI_ (AUC ≥ 0.92, *p* < 0.001) performed considerably better than ARNFLT, Mag, and MagD (AUC ≤ 0.81, *p* ≥ 0.003) in distinguishing GS from PPG eyes (Table 6).

**Table 6:** Results from ROC curve analysis among study participants

	Healthy eyes vs. GS eyes		GS eyes vs. PPG eyes	
	Area	*p*-value	Area	*p*-value
ARNFLT*^†^	0.89	< 0.001	0.81	0.003
Mag*	0.81	< 0.001	0.58	0.57
MagD*^†^	0.80	< 0.001	0.76	0.018
eRGC_CSFI_ *^†^	0.85	< 0.001	0.98	< 0.001
eRGC_Mag_*^†^	0.77	0.006	0.92	< 0.001
eRGC_MagD_*^†^	0.78	0.004	0.94	< 0.001

*ARNFLT,* average retinal nerve fiber layer thickness (μM); *Mag* transformed magnitude (μV); *MagD* transformed magnitudeD (μV); *RGC*_*CSFI*_ retinal ganglion cell count estimated with the combined structure–function index; *eRGC*_*Mag*_ retinal ganglion cell count estimated with Mag and ARNFLT*; eRGC*_*MagD*_ retinal ganglion cell count estimated with MagD and ARNFLT

* p < *0.05* for control eyes vs. glaucoma suspect (GS) eyes.^†^* p* < *0.05* for GS eyes vs. preperimetric glaucoma (PPG) eyes

**Fig. 2:**
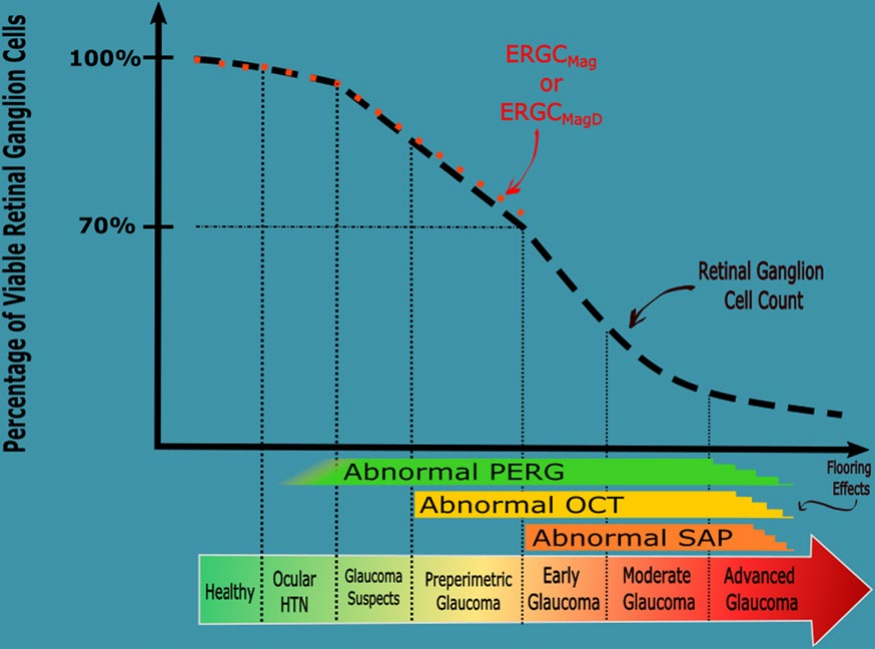
Composite image of Cirrhus OCT, Diopsys ssPERG, and HVF 24–2 data in both eyes of one healthy subject. Hc: High-contrast; Lc: Low-contrast

**Fig. 3:**
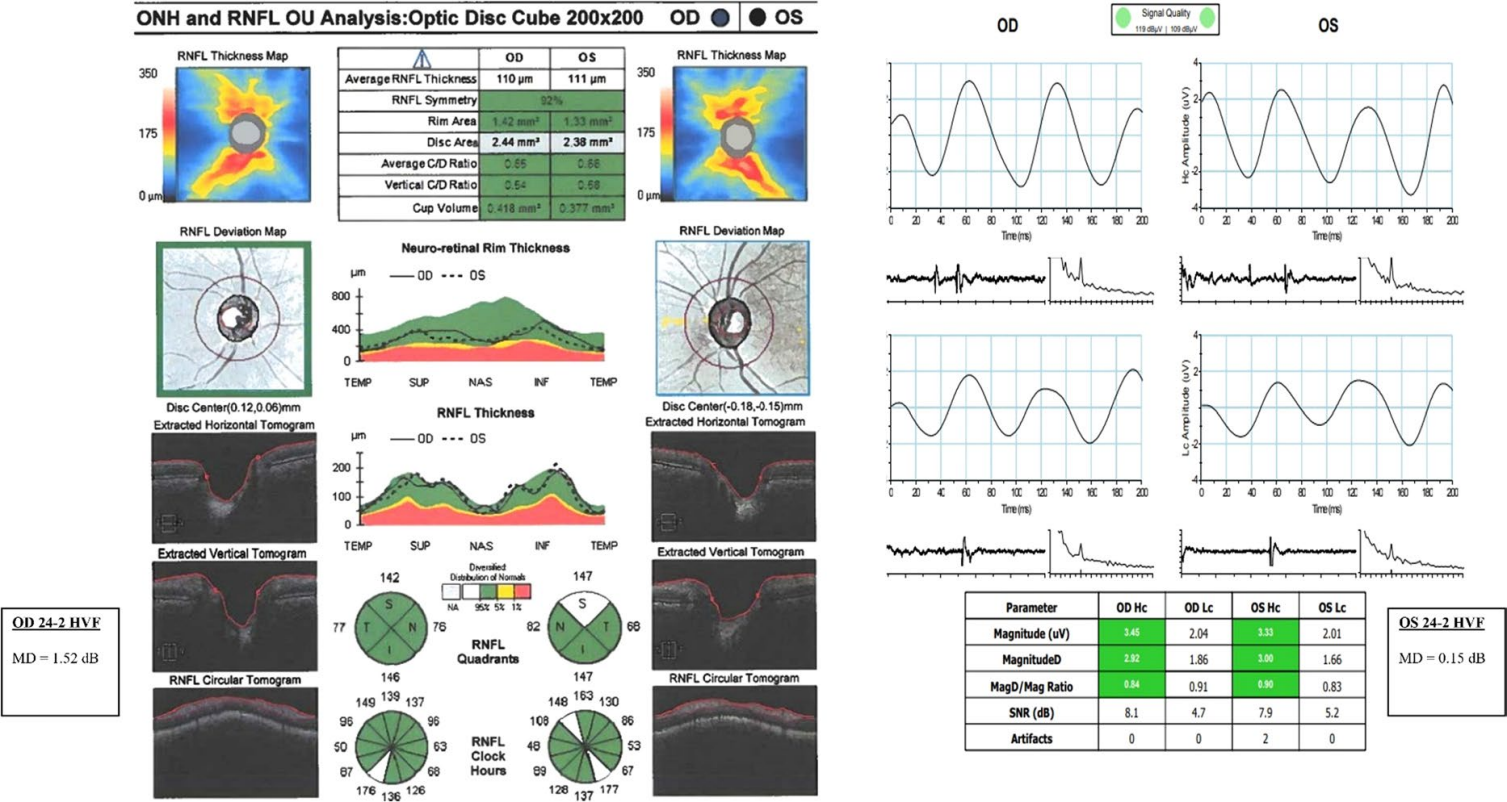
Composite image of Cirrhus OCT, Diopsys ssPERG, and HVF 24–2 data in both eyes of one glaucoma suspect. *Hc* High-contrast; *Lc* Low-contrast

The average eRGC_Mag_ was 1,068,581 in healthy subjects, 924,701 in GS, and 723,352 in PPG eyes (Table 7). The average eRGC_MagD_ was 1,070,825 in healthy subjects, 927,134 in GS, and 718,722 in PPG, which are similar to eRGC_CSFI_ values seen in previous studies [36]. In GS, ssPERG parameter losses (31–37%) were proportionally higher than estimated RGC losses (13%) and ARNFLT losses (12%). In PPG, Mag losses (32%) and estimated RGC losses (36%) were similar, whereas MagD losses were high (54%) and ARNFLT losses were relatively low (18%). Both GLMMs estimated age-related RGC losses at rate of 0.57–0.59% per year (Table 7). Examples of OCT, ssPERG, and 24-2 MD values for healthy eyes and GS eyes can be found in Figs. 2 and 3, respectively.

**Table 7:** Proportional losses of structural and functional parameters, and estimated retinal ganglion cell count, in GS (*N* = 10) and PPG (*N* = 10) eyes relative to healthy (*N* = 30) eyes

	Healthy eyes	GS eyes (% Loss)	PPG eyes (% Loss)
eRGC_Mag_	1,068,581	924,701 (13%)	723,352 (32%)
eRGC_MagD_	1,070,825	927,134 (13%)	718,722 (33%)
eRGC_Mag_ per year	–	6268 (0.59%)	6268 (0.59%)
eRGC_MagD_ per year	–	6092 (0.57%)	6092 (0.57%)
ARNFLT (μM)	96.80	85.00 (12%)	79.20 (18%)
Mag (μV)	1.95	1.35 (31%)	1.25 (36%)
MagD (μV)	1.72	1.09 (37%)	0.79 (54%)

*eRGC*_*Mag*_*,* retinal ganglion cell count estimated with Mag and ARNFLT; *eRGC*_*MagD*_ retinal ganglion cell count estimated with MagD and ARNFLT*; ARNFLT* average retinal nerve fiber layer thickness; *Mag* magnitude; *MagD* magnitudeD

## Discussion

The purpose of this pilot study was to determine whether ssPERG and OCT parameters could be used to estimate RGC count in GS and PPG. As a large body of literature provides evidence that PERG parameters directly measure RGC activity [7–9, 12, 17, 29, 37–39], it is reasonable to hypothesize that ssPERG is capable of estimating RGC count within a combined structure–function model. However, to the best of our knowledge there have been no attempts to estimate RGC count with ssPERG until the current study. We demonstrated that two structure–function GLMMs were capable of accurately predicting eRGC_CSFI_ from age, ARNFLT, and ssPERG parameters, and the accuracy of these models was validated through cross-validation [40, 41].

The GLMM is a maximum likelihood-based approach to regression statistics developed to predict an outcome from prediction variables that are correlated [32, 33, 42]. This makes GLMMs particularly useful when both eyes from study subjects are analyzed, as outcomes can be biased by within-subject inter-eye correlations [33, 43]. In fact, GLMMs have effectively accounted for such correlations when predicting continuous outcomes in cross-sectional studies [33]. To reduce the effect of multicollinearity, we elected to create two separate models for Mag and MagD, rather than a single model that utilized both ssPERG parameters [40].

The assumption of linearity between outcome and predictive measures was supported in this study by eRGC_CSFI_ significantly associating with Mag, MagD, age, and ARNFLT (Table 2, *p* < 0.001) [44], and these correlations agree with findings in our previous study [18]. Both GLMMs demonstrated significant equations (*p* < 0.001 for both models), with all prediction values contributing significantly to each model (*p* ≤ 0.027) (Table 3). Importantly, *R*^2^ values from the training group were highly similar to the *R*^2^ values from the validation group (*R*^2^ = 0.90 and 0.89, respectively, for both GLMMs) (Fig. 1), which supports the external validity of these models [40]. Interestingly, the GLMMs estimated age-related RGC losses at a rate of 0.57–0.59% per year (Table 7). These losses were remarkably similar to at least five studies that used similar regression statistics to estimate age-related losses at a rate of 0.55–0.61% per year [31, 45–48], suggesting that both models accurately accounted for age-related RGC losses. Post hoc multivariate pairwise comparisons revealed that eRGC_Mag_ and eRGC_MagD_ distinguished between healthy, GS, and PPG eyes (*p* < 0.001 for all comparisons), whereas independent ssPERG measures and ARNFLT could not distinguish between GS and PPG eyes (Table 5). Clinically, this suggests that combining ssPERG and SD-OCT parameters into a unified metric may distinguish between GS and PPG eyes better than independent ssPERG and SD-OCT measurements.

Previous experimental studies investigating RGC losses in glaucoma have consistently demonstrated a nonlinear relationship between RGC losses and 24–2 MD losses, with large RGC losses corresponding to small changes in 24–2 MD in early disease [2, 8, 49]. In fact, multiple studies showed that 25–50% of RGCs were lost before vision loss was identifiable with SAP [48, 50–52]. This relationship between RGC count and SAP measures is likely attributable to the logarithmic scale SAP utilizes to quantify vision loss, which can deflate subtle deficits in early disease [53]. Experimental studies found that utilizing linear units to assess visual acuity resulted in a linear relationship between RGC functional losses and visual field losses, especially when age-related RGC losses and retinal eccentricity were also considered [8, 52]. The linear relationship between RGC losses and visual deficits have been further supported by ssPERG studies, which have linearly correlated RGC dysfunction with eRGC_CSFI_ losses, and with SAP sensitivity losses before logarithmic transformations [8, 18]. Taken together, these findings support the hypothesis that vision loss occurs concurrently with RGC losses in glaucoma, with an apparent nonlinear relationship attributable to limitations in SAP testing.

The results of this study further support this hypothesis by demonstrating no significant mean differences in 24–2 MD among study groups despite significant mean differences in estimated RGC count (Table 5). Furthermore, estimated RGC count in PPG eyes was 32–33% less than estimates in healthy eyes (Table 7), which suggests that 24-2 MD in PPG eyes was not significantly lower than healthy eyes despite a 32–33% decrease in estimated RGC count. Importantly, a 32–33% decrease in RGC count is similar to, or less than the threshold for significant visual field losses observed in prior investigations [48, 50–52].

The results of this study also support the hypothesis that dysfunctional but viable RGCs are present in GS and PPG eyes. As discussed in detail in our previous study, Mag is likely a measure of overall RGC death and/or dysfunction, whereas MagD is adjusted to account for RGC downstream signaling delays that likely result from pathological changes in RGC morphology [18]. Ventura et al. demonstrated that PERG losses in GS exceeded the proportion expected from ARNFLT losses [54], and follow-up studies found significantly higher rates of signaling delays in the earliest stages of glaucoma when compared to healthy controls despite a constant rate of overall RGC functional losses [7]. These data strongly suggest that advancing stages of glaucoma result in a decrease in overall RGC function and an increase in RGC signaling delays.

In this study, we found Mag and MagD to be 31% and 37% lower in GS relative to healthy eyes, respectively, which were much larger losses than expected from a 12% loss in ARNFLT and 13% difference in estimated RGC count (Table 7). In PPG eyes, however, Mag losses and estimated RGC losses were similar (36 vs. 32–33%, respectively), whereas MagD losses increased considerably (54% loss) and ARNFLT losses increased less appreciably (18% loss). These results suggest that a proportion of RGCs begin to lose functionality *before* cell death in GS, as demonstrated by a higher percent loss of Mag than estimated RGC count. As the disease progresses to PPG, *dysfunctional RGCs are lost*, as demonstrated by a similar proportional loss of Mag and estimated RGC count. As RGCs undergo cell death, changes in RGC morphology affect the temporal dynamics of RGC neuronal pathways and result in signaling delays, as demonstrated by a large proportional loss of MagD in PPG eyes (54% loss). These pathological changes in RGCs have a small effect on ARNFLT in early disease, as demonstrated by an 18% loss in PPG eyes. Such findings agree with previous studies that found PERG losses preceding equivalent ARNFLT losses by about 8 years [55].

For example, Fig. 3 is a composite image of OCT, ssPERG, and 24–2 HVF results in two GS eyes from one study subject. Both eyes have an ARNFLT on the lower limit of normal adjusted for age, and both eyes also have concern for superior quadrant defects. The left eye has an increased cup-to-disc ratio and a severe defect in the 11 o-clock clock hour sector, which suggests that the left eye has more severe structural damage. However, ssPERG and 24–2 HVF data suggest that the right eye has more severe functional loss. Conceptually, estimating RGC count through GLMMs may offer a new approach to distinguishing GS from glaucoma patients by combining structural and functional parameters into highly sensitive and specific RGC estimates (Table 6). Future longitudinal studies with a larger cohort of study participants are warranted to investigate this hypothesis before such recommendations can be considered.

**Fig. 4:**
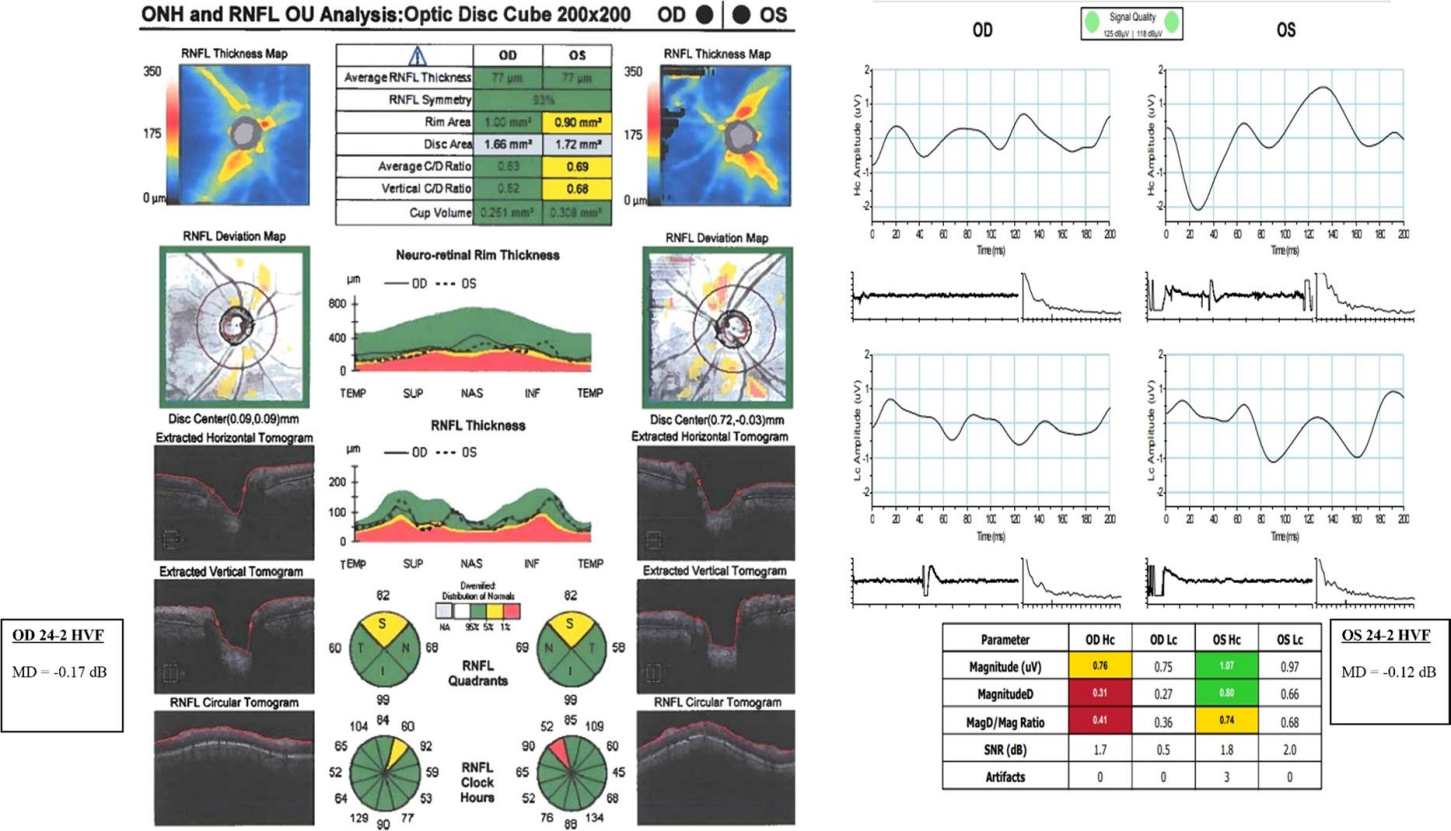
Conceptual diagram illustrating the structure–function relationship through advancing stages of glaucoma. Healthy subjects have normal PERG (green), OCT (yellow), and SAP (orange) findings, with a negligible age-related decline in RGC count (black, dotted line). Ocular hypertension can induce oxidative stress RGCs, which may cause abnormal PERG findings in some individuals. Pathological RGC losses begin in glaucoma suspects with associated abnormal PERG. Rapidly declining RGC count in borderline and early glaucoma contributes to retinal thinning, resulting in abnormal OCT. Abnormal SAP is seen after ~ 40% of RGCs are lost. PPG eyes are characterized by normal SAP measures with abnormal OCT and ssPERG findings. In severe glaucoma, flooring effects occur in OCT, PERG, and SAP. Retinal ganglion cell count estimated from the models in this study (eRGC_Mag_ or eRGC_MagD_, red dotted line) decline linearly from the onset of PERG losses through to PPG. Conceptually, eRGC_Mag_ or eRGC_MagD_ may offer a single quantifiable metric for discerning between healthy, GS, and PPG eyes

Here, it is important to acknowledge that our models were derived from the CSFI, which in and of itself does not represent a “true” RGC count. As discussed by Raza and Hood, the Harwerth model for estimating RGC count [52], which is the foundation for the CSFI, overestimated RGC count in humans postmortem [18, 56]. However, the CSFI is geared more to predicting glaucomatous progression through a unified structure–function measurement, rather than obtaining highly accurate RGC count estimates [20]. In the same context, we do not intend for eRGC_Mag_ and eRGC_MagD_ to be interpreted as a “true” RGC count, but instead to demonstrate how ssPERG and SD-OCT measures can be unified through linear mixed modeling to estimate RGC count.. For example, Fig. 4 is a conceptual diagram illustrating the relationships among structural and functional measurements, and RGC count, through advancing stages of glaucoma. Healthy eyes have no clinically identifiable deficits in ssPERG, OCT, or SAP. Ocular hypertension (OHTN) is characterized by the absence of clinical and morphological deficits despite elevated IOP [57], although some studies provided evidence that OHTN may be associated with abnormal PERG [58, 59]. In GS, several studies have demonstrated reduced ssPERG parameters in the absence of OCT and SAP losses [11, 55]. Eventually, RGC losses result in retinal thinning, which is identified clinically with OCT [18]. Eyes with abnormal OCT withoutSAP losses are commonly staged as PPG eyes [60], and when SAP becomes severely abnormal, glaucoma is diagnosed [61]. By the point when 24–2 MD becomes significantly low, studies found that at least 25–35% of RGCs are already irreversibly lost [48], and the results of this study suggest that as high as a 32–33% loss of eRGC_Mag_ did not significantly reduce 24–2 MD (Tables 5, 7). As such, identifying which GS eyes are progressing to PPG is paramount to disease control and improving life quality [62]. However, only 9.5% of GS progress to glaucoma [63], and determining which eyes are at the highest risk can be difficult, particularly when IOP, SAP, OCT, and ssPERG measures are normal or in conflict.

The present study had limitations. The number of eyes per group were unequal and the equality of variance was not assumed. Therefore, this study utilized Games-Howell post hoc analyses to account for unequal variance [35]. Unlike eRGC_CSFI_, which was derived to address all stages of glaucoma [19], eRGC_Mag_ and eRGC_MagD_ are limited to GS and PPG (Fig. 4). However, eRGC_Mag_ and eRGC_MagD_ do have several advantages within these stages. eRGC_Mag_ and eRGC_MagD_ can be obtained using a single formula that requires only 3 data points per eye, which, when compared to the 9 equations and 57 data points per eye required to generate eRGC_CSFI_ [19], may offer clinicians a more efficient approach to estimating RGC count. Additionally, ssPERG was found to detect functional losses before SAP in glaucoma [11, 13, 14], and demonstrated good within-subject and between-trial repeatability when utilizing PERGLA protocol [13]. When compared to a high test–retest variability in SAP [64, 65], utilizing ssPERG in lieu of SAP may allow eRGC_Mag_ and eRGC_MagD_ to yield more consistent results in early disease. In fact, the limitations of SAP measures in early glaucoma are well described [2, 52], and one study found that focal visual deficits may be detectable with SAP up to one year earlier if test–retest variability was reduced by 30–60% [66]. Our study was also limited by a relatively small sample size, and age was also significantly different amongst study groups (Table 1). However, the GLMMs used to estimate RGC count in this study did not take glaucoma grouping into account and were instead dependent on training and validation grouping. Importantly, age was not significantly different between the training and validation groups (Table 3). Nonetheless, future studies that utilize a larger cohort with an equal number of patients in each group and non-significant differences in age are warranted. Finally, our models are limited by the availability of ssPERG systems in a clinical setting. Any clinician or academic scientist seeking to estimate RGC count with our models would need to have access to a ssPERG system and follow the PERGLA protocol to ensure measurements consistent with those used in this study.

## Conclusion

The current study presents the first combined structure–function models for estimating RGC count using ssPERG and OCT parameters. Estimating RGC count with these models yielded similar estimates to those estimated with the CSFI and performed better than independent ssPERG and OCT measures in distinguishing healthy, GS, and PPG eyes. Estimates generated from these models are generalizable to healthy, GS, and PPG subjects.

## Abbreviations

ARNFLT: Average retinal nerve fiber layer thickness
CSFI: Combined structure–function index
eRGC_CSFI_: Combined structure–function index estimated retinal ganglion cell count
eRGC_Mag_: Magnitude estimated retinal ganglion cell count
eRGC_MagD_: MagnitudeD estimated retinal ganglion cell count
GHT: Glaucoma hemifield test
GLMM: Generalized linear mixed model
GS: Glaucoma suspect
Mag: Magnitude
MagD: MagnitudeD
MD: Mean deviation
OCT: Optical coherence tomography
PPG: Preperimetric glaucoma
RGC: Retinal ganglion cell
SAP: Standard automated perimetry
SD-OCT: Spectral-domain optical coherence tomography
ssPERG: Steady-state pattern electroretinography

## Declarations

### Statement on the welfare of animals

This study did not involve the use of animal subjects.
